# Supplementary material for: Modeling glioblastoma heterogeneity as a dynamic network of cell states
Source: Mol Syst Biol. 2021 Sep 16;17(9):e10105. doi: 10.15252/msb.202010105 (PMC8444284; doi:10.15252/msb.202010105)
Supplement: Supplementary file 5 — Source Data for Figure 3 [file MSB-17-e10105-s001.zip › Figure3A_sourcedata/GSEA_3065/hallmarks_state6.GseaPreranked.1623416308702/HALLMARK_EPITHELIAL_MESENCHYMAL_TRANSITION.html]

Details for gene set HALLMARK\_EPITHELIAL\_MESENCHYMAL\_TRANSITION[GSEA]

|  || Dataset | state6 |
| Phenotype | NoPhenotypeAvailable |
| Upregulated in class | na\_neg |
| GeneSet | HALLMARK\_EPITHELIAL\_MESENCHYMAL\_TRANSITION |
| Enrichment Score (ES) | -0.6700412 |
| Normalized Enrichment Score (NES) | -2.775269 |
| Nominal p-value | 0.0 |
| FDR q-value | 0.0 |
| FWER p-Value | 0.0 |
Table: GSEA Results Summary

  

Fig 1: Enrichment plot: HALLMARK\_EPITHELIAL\_MESENCHYMAL\_TRANSITION      
 Profile of the Running ES Score & Positions of GeneSet Members on the Rank Ordered List

  

| PROBE | GENE SYMBOL | GENE\_TITLE | RANK IN GENE LIST | RANK METRIC SCORE | RUNNING ES | CORE ENRICHMENT || 1 | GADD45A |  |  | 2 | 0.419 | 0.0294 | No |
| 2 | DPYSL3 |  |  | 464 | 0.127 | -0.0147 | No |
| 3 | SDC1 |  |  | 698 | 0.106 | -0.0340 | No |
| 4 | SAT1 |  |  | 744 | 0.102 | -0.0320 | No |
| 5 | PFN2 |  |  | 942 | 0.089 | -0.0484 | No |
| 6 | ID2 |  |  | 1058 | 0.082 | -0.0558 | No |
| 7 | FGF2 |  |  | 1185 | 0.076 | -0.0649 | No |
| 8 | TPM4 |  |  | 1285 | 0.072 | -0.0712 | No |
| 9 | SLC6A8 |  |  | 1391 | 0.068 | -0.0785 | No |
| 10 | GADD45B |  |  | 1497 | 0.064 | -0.0861 | No |
| 11 | MEST |  |  | 1882 | 0.051 | -0.1267 | No |
| 12 | MYLK |  |  | 2302 | 0.038 | -0.1723 | No |
| 13 | PMEPA1 |  |  | 2388 | 0.036 | -0.1795 | No |
| 14 | TGFBI |  |  | 2809 | 0.026 | -0.2260 | No |
| 15 | SFRP1 |  |  | 2812 | 0.026 | -0.2244 | No |
| 16 | VIM |  |  | 2831 | 0.026 | -0.2246 | No |
| 17 | FN1 |  |  | 2839 | 0.026 | -0.2236 | No |
| 18 | SDC4 |  |  | 3129 | 0.019 | -0.2555 | No |
| 19 | FUCA1 |  |  | 3169 | 0.018 | -0.2587 | No |
| 20 | PLOD1 |  |  | 3255 | 0.016 | -0.2674 | No |
| 21 | TPM1 |  |  | 3680 | 0.007 | -0.3157 | No |
| 22 | QSOX1 |  |  | 3763 | 0.005 | -0.3248 | No |
| 23 | SERPINE1 |  |  | 3811 | 0.004 | -0.3299 | No |
| 24 | APLP1 |  |  | 3813 | 0.004 | -0.3297 | No |
| 25 | SNTB1 |  |  | 3888 | 0.003 | -0.3380 | No |
| 26 | PLAUR |  |  | 3956 | 0.001 | -0.3456 | No |
| 27 | TPM2 |  |  | 4007 | 0.001 | -0.3514 | No |
| 28 | MSX1 |  |  | 4097 | -0.001 | -0.3615 | No |
| 29 | SERPINE2 |  |  | 4250 | -0.004 | -0.3787 | No |
| 30 | MCM7 |  |  | 4435 | -0.008 | -0.3994 | No |
| 31 | CAP2 |  |  | 4567 | -0.010 | -0.4138 | No |
| 32 | ITGAV |  |  | 4722 | -0.013 | -0.4306 | No |
| 33 | NOTCH2 |  |  | 4874 | -0.016 | -0.4469 | No |
| 34 | CAPG |  |  | 4878 | -0.016 | -0.4461 | No |
| 35 | TIMP3 |  |  | 5017 | -0.019 | -0.4606 | No |
| 36 | TAGLN |  |  | 5323 | -0.025 | -0.4940 | No |
| 37 | LAMA1 |  |  | 5340 | -0.025 | -0.4941 | No |
| 38 | VEGFA |  |  | 5660 | -0.032 | -0.5286 | No |
| 39 | THBS1 |  |  | 5670 | -0.032 | -0.5274 | No |
| 40 | PDLIM4 |  |  | 5808 | -0.035 | -0.5407 | No |
| 41 | WIPF1 |  |  | 5838 | -0.035 | -0.5416 | No |
| 42 | ENO2 |  |  | 5889 | -0.036 | -0.5448 | No |
| 43 | BDNF |  |  | 6042 | -0.039 | -0.5595 | No |
| 44 | CXCL12 |  |  | 6173 | -0.042 | -0.5715 | No |
| 45 | COPA |  |  | 6322 | -0.045 | -0.5854 | No |
| 46 | GAS1 |  |  | 6366 | -0.046 | -0.5871 | No |
| 47 | FERMT2 |  |  | 6508 | -0.050 | -0.5998 | No |
| 48 | ITGA5 |  |  | 6637 | -0.054 | -0.6107 | No |
| 49 | BMP1 |  |  | 6662 | -0.054 | -0.6096 | No |
| 50 | TGFB1 |  |  | 6712 | -0.056 | -0.6113 | No |
| 51 | NNMT |  |  | 6762 | -0.058 | -0.6128 | No |
| 52 | GJA1 |  |  | 6876 | -0.061 | -0.6215 | No |
| 53 | MMP2 |  |  | 7016 | -0.065 | -0.6329 | No |
| 54 | GLIPR1 |  |  | 7136 | -0.069 | -0.6418 | No |
| 55 | PTHLH |  |  | 7156 | -0.070 | -0.6390 | No |
| 56 | WNT5A |  |  | 7276 | -0.074 | -0.6475 | No |
| 57 | RHOB |  |  | 7306 | -0.075 | -0.6456 | No |
| 58 | COL16A1 |  |  | 7352 | -0.076 | -0.6454 | No |
| 59 | COL5A3 |  |  | 7415 | -0.078 | -0.6470 | No |
| 60 | PRRX1 |  |  | 7482 | -0.081 | -0.6489 | No |
| 61 | JUN |  |  | 7513 | -0.082 | -0.6465 | No |
| 62 | PLOD2 |  |  | 7718 | -0.092 | -0.6635 | Yes |
| 63 | LRP1 |  |  | 7748 | -0.093 | -0.6603 | Yes |
| 64 | FBN1 |  |  | 7777 | -0.094 | -0.6569 | Yes |
| 65 | TNFRSF12A |  |  | 7784 | -0.094 | -0.6509 | Yes |
| 66 | ITGB5 |  |  | 7832 | -0.098 | -0.6494 | Yes |
| 67 | SGCB |  |  | 7845 | -0.098 | -0.6438 | Yes |
| 68 | FBLN1 |  |  | 7857 | -0.099 | -0.6381 | Yes |
| 69 | EFEMP2 |  |  | 7869 | -0.099 | -0.6323 | Yes |
| 70 | CDH11 |  |  | 7885 | -0.101 | -0.6269 | Yes |
| 71 | MATN2 |  |  | 7984 | -0.106 | -0.6307 | Yes |
| 72 | LOXL1 |  |  | 8154 | -0.117 | -0.6418 | Yes |
| 73 | FLNA |  |  | 8171 | -0.119 | -0.6353 | Yes |
| 74 | HTRA1 |  |  | 8245 | -0.126 | -0.6348 | Yes |
| 75 | COL6A2 |  |  | 8253 | -0.126 | -0.6267 | Yes |
| 76 | CADM1 |  |  | 8282 | -0.130 | -0.6207 | Yes |
| 77 | SPARC |  |  | 8303 | -0.132 | -0.6136 | Yes |
| 78 | PTX3 |  |  | 8319 | -0.134 | -0.6059 | Yes |
| 79 | FSTL1 |  |  | 8373 | -0.143 | -0.6018 | Yes |
| 80 | NT5E |  |  | 8380 | -0.144 | -0.5923 | Yes |
| 81 | COL5A1 |  |  | 8428 | -0.150 | -0.5871 | Yes |
| 82 | THY1 |  |  | 8454 | -0.153 | -0.5792 | Yes |
| 83 | COLGALT1 |  |  | 8471 | -0.155 | -0.5701 | Yes |
| 84 | GPC1 |  |  | 8473 | -0.155 | -0.5592 | Yes |
| 85 | CD59 |  |  | 8508 | -0.163 | -0.5516 | Yes |
| 86 | COL5A2 |  |  | 8514 | -0.165 | -0.5405 | Yes |
| 87 | COL11A1 |  |  | 8518 | -0.165 | -0.5292 | Yes |
| 88 | COL4A2 |  |  | 8525 | -0.168 | -0.5180 | Yes |
| 89 | ADAM12 |  |  | 8526 | -0.168 | -0.5061 | Yes |
| 90 | P3H1 |  |  | 8547 | -0.172 | -0.4962 | Yes |
| 91 | PMP22 |  |  | 8560 | -0.175 | -0.4852 | Yes |
| 92 | GPX7 |  |  | 8596 | -0.189 | -0.4759 | Yes |
| 93 | NTM |  |  | 8612 | -0.196 | -0.4637 | Yes |
| 94 | IGFBP3 |  |  | 8615 | -0.197 | -0.4500 | Yes |
| 95 | LAMC1 |  |  | 8618 | -0.199 | -0.4362 | Yes |
| 96 | EMP3 |  |  | 8630 | -0.203 | -0.4231 | Yes |
| 97 | PCOLCE |  |  | 8639 | -0.209 | -0.4093 | Yes |
| 98 | DST |  |  | 8644 | -0.211 | -0.3948 | Yes |
| 99 | SERPINH1 |  |  | 8646 | -0.212 | -0.3799 | Yes |
| 100 | CALD1 |  |  | 8649 | -0.213 | -0.3651 | Yes |
| 101 | COL4A1 |  |  | 8661 | -0.218 | -0.3509 | Yes |
| 102 | VCAN |  |  | 8664 | -0.219 | -0.3356 | Yes |
| 103 | LOXL2 |  |  | 8687 | -0.232 | -0.3217 | Yes |
| 104 | PLOD3 |  |  | 8693 | -0.236 | -0.3056 | Yes |
| 105 | CDH2 |  |  | 8695 | -0.237 | -0.2889 | Yes |
| 106 | IGFBP2 |  |  | 8719 | -0.260 | -0.2732 | Yes |
| 107 | DKK1 |  |  | 8749 | -0.288 | -0.2562 | Yes |
| 108 | TIMP1 |  |  | 8751 | -0.290 | -0.2358 | Yes |
| 109 | TFPI2 |  |  | 8752 | -0.290 | -0.2153 | Yes |
| 110 | ITGB1 |  |  | 8777 | -0.335 | -0.1943 | Yes |
| 111 | CD44 |  |  | 8778 | -0.337 | -0.1705 | Yes |
| 112 | SPP1 |  |  | 8779 | -0.338 | -0.1466 | Yes |
| 113 | PPIB |  |  | 8782 | -0.351 | -0.1219 | Yes |
| 114 | CALU |  |  | 8784 | -0.362 | -0.0964 | Yes |
| 115 | TNC |  |  | 8787 | -0.389 | -0.0692 | Yes |
| 116 | SCG2 |  |  | 8793 | -0.455 | -0.0375 | Yes |
| 117 | POSTN |  |  | 8795 | -0.533 | 0.0001 | Yes |
Table: GSEA details [plain text format]

  

Fig 2: HALLMARK\_EPITHELIAL\_MESENCHYMAL\_TRANSITION: Random ES distribution      
 Gene set null distribution of ES for **HALLMARK\_EPITHELIAL\_MESENCHYMAL\_TRANSITION**
